# Surveillance of Antimicrobial Resistance in *Neisseria gonorrhoeae* in Alberta from 2016–2022

**DOI:** 10.3390/antibiotics14111119

**Published:** 2025-11-06

**Authors:** Taylor M. Walsh, Sabrina S. Plitt, Tanis C. Dingle, Carmen L. Charlton

**Affiliations:** 1 Department of Laboratory Medicine and Pathology, University of Alberta, Edmonton, AB T6G 1C9, Canada; 2 Women and Children’s Health Research Institute (WCHRI), University of Alberta, Edmonton, AB T6G 1C9, Canada; 3 School of Public Health, University of Alberta, Edmonton, AB T6G 1C9, Canada; 4 Provincial Public Health Laboratory, Alberta Precision Laboratories, Calgary, AB T2N 1M7, Canada; 5 Department of Pathology and Laboratory Medicine, University of Calgary, Calgary, AB T2N 1N4, Canada; 6 Provincial Public Health Laboratory, Alberta Precision Laboratories, Edmonton, AB T6G 2J2, Canada; 7 Li Ka Shing Institute of Virology, University of Alberta, Edmonton, AB T6G 2E1, Canada; 8 Canadian Blood Services, Surveillance and Discovery Laboratory, Ottawa, ON K1G 4J5, Canada; 9 Canadian Blood Services, Donation Policy and Studies, Edmonton, AB T6G 2R8, Canada

**Keywords:** antimicrobial susceptibility testing, *Neisseria gonorrhoeae*, antimicrobial resistance, surveillance, trend analysis, gonorrhea

## Abstract

Background/Objectives: *Neisseria gonorrhoeae* can develop resistance to antimicrobial treatments, posing a challenge to effective management of patients. Alberta, Canada, monitors the antimicrobial susceptibility of gonorrhea isolates to track resistance trends. This study aims to retrospectively analyze susceptibility data and demographic trends from gonorrhea cases in the province over a seven-year period. Methods: Antimicrobial susceptibility testing was performed using gradient strip methodology on gonorrhea isolates from Alberta, evaluating both historical and currently recommended antimicrobials for treatment of gonorrhea. Susceptibility testing results were interpreted using Clinical and Laboratory Standards Institute (CLSI) breakpoints. Provincial antimicrobial susceptibility testing data were analyzed using STATA v.17, incorporating antimicrobial resistance patterns and demographic information from provincial databases. Results: Between 2016 and 2022, 4056 *N. gonorrhoeae* isolates were cultured from 3617 individuals. All isolates tested were susceptible to ceftriaxone and cefixime, except for a single resistant isolate in 2018. Azithromycin susceptibility ranged from 99% to 88%, with the lowest susceptibility observed in 2018. Males exhibited higher rates of antimicrobial non-susceptibility than females across all drugs tested, except for tetracycline. Conclusions: Ongoing antimicrobial susceptibility surveillance in Alberta is crucial for identifying resistance trends and informing the development of effective treatment strategies for gonorrhea.

## 1. Introduction

*Neisseria gonorrhoeae* is a bacterial pathogen responsible for infection of mucous membranes in the genitals, rectum, throat, and eyes in both males and females [1]. In symptomatic infections, *N. gonorroheae* can cause urethral stricture, urogenital tract abscesses, prostatitis, and epididymo-orchitis in men, and cervicitis or urethritis and pelvic inflammatory disease in women [2]. Disseminated gonococcal infection occurs particularly in immunocompromised individuals, and can result in septic arthritis, arthralgias, tenosynovitis, and multiple skin lesions [3]. However, infection can also be asymptomatic, with approximately 20% of males and 50% of females presenting with no observable symptoms. This high asymptomatic rate can complicate public health efforts to reduce transmission [4]. Several factors increase the risk of *N. gonorrhoeae* infection, including a higher number of lifetime sexual partners, casual sex, lower educational levels, unprotected intercourse, age under 25 years, a history of sexually transmitted and blood-borne infections (STBBIs), and engaging in sex for money or drugs [5,6].

Penicillin was introduced as the first-line treatment for gonorrhea in 1943 and was widely effective for decades. However, by 1967, rising resistance to penicillin was evident [7]. Tetracycline, used as an alternative for penicillin-allergic patients since the 1940s, also saw high levels of resistance by 1985 [8]. These growing resistance patterns led the Centers for Disease Control and Prevention (CDC) to remove both penicillin and tetracycline from the list of recommended treatments by 1989 [9,10]. Ciprofloxacin, introduced in the mid-1980s, initially appeared promising, but by the 1990s, resistant strains began to emerge, and by 2007, it was no longer recommended as a first-line treatment [9,11].

Until recently, in Canada, the treatment regimen for *N. gonorrhoeae* infection, established in 2013, recommended dual therapy with either cefixime and azithromycin or ceftriaxone and azithromycin [12,13,14]. In 2025, the Canadian treatment recommendations were updated due to growing concerns of azithromycin resistance and now recommend a single intramuscular dose of ceftriaxone as the preferred treatment option for uncomplicated gonococcal infections [13]. Despite these changes, concerns about treatment failures have emerged [15]. The World Health Organization (WHO) has reported cases of treatment failure with both ceftriaxone and cefixime [16], with Japan documenting marked decreased susceptibility to cefixime since 2001 [17], and the United Kingdom observing a dual therapy failure in 2016 [18]. In Alberta, a treatment failure with ceftriaxone and azithromycin was reported in 2018, with the strain exhibiting genetic alterations similar to those found in a 2015 strain from Japan [19,20]. These cases highlight the growing concern that decreasing antimicrobial susceptibility may soon outpace available treatment options.

In response to these challenges, the WHO established the Gonococcal Antimicrobial Susceptibility Programme (GASP) to monitor *N. gonorrhoeae* resistance trends and inform global, national, and local public health strategies [21]. Canada is a participating country in this program. In 2021, Canada tested 3439 *N. gonorrhoeae* isolates for antimicrobial resistance (AMR), finding a rise in resistance to cefixime (1.5% in 2021 vs. 0.6% in 2017), while resistance to ceftriaxone remained low [22]. While azithromycin resistance decreased, an increasing number of isolates showed higher minimum inhibitory concentrations (MICs) than in previous years [22].

In Alberta, AMR surveillance for *N. gonorrhoeae* has been conducted since 2001. The most recent data (2012–2016) revealed no resistance to ceftriaxone, though a small number of isolates showed decreased susceptibility to cefixime in 2014 [23]. Azithromycin resistance remained low, fluctuating between 0.4% and 1.8%, with no clear temporal trends [23].

Here, we examine current antimicrobial susceptibility testing (AST) results for *N. gonorrhoeae* isolates in Alberta from 2016 to 2022 and examine demographic trends to understand AMR patterns in the province’s population. By examining provincial surveillance data, these results can be used to understand AMR trends in our local population and can be used to inform future public health interventions.

## 2. Results and Discussion

**Figure 1:**
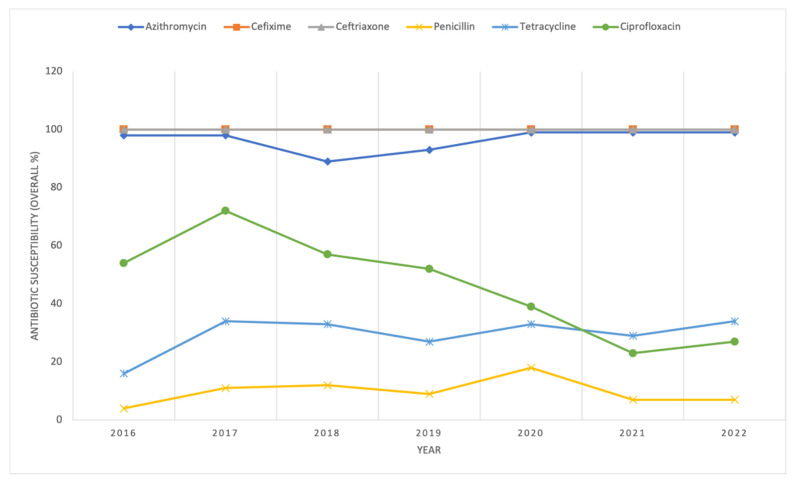
*N. gonorrhoeae* susceptibility by year in culture-positive isolates in Alberta, Canada, between 2016 and 2022. Antibiotic susceptibilities for penicillin, tetracycline, ciprofloxacin, cefixime, azithromycin, and ceftriaxone calculated by year (the number of susceptible isolates divided by the total number of isolates tested).

Duplicate cultures submitted on the same day were removed from the database, resulting in 4056 *N. gonorrhoeae* isolates available for analysis between 2016 and 2022. Not all antimicrobials were tested for each isolate. Among the antibiotics tested, penicillin exhibited the lowest susceptibility across all years, followed by tetracycline and ciprofloxacin, which, until 2021, had higher susceptibility than tetracycline (Figure 1). All isolates were susceptible to ceftriaxone and cefixime, except in 2018, where a single isolate showed resistance to both antimicrobials (susceptibility: 840/841, 99.9%). Azithromycin susceptibility remained relatively stable over time, with the lowest observed in 2018 at 88% (745/842). In 2022, 100% of isolates (391) were susceptible to ceftriaxone and cefixime, 99% (386) to azithromycin, 34% (131) to tetracycline, 27% (106) to ciprofloxacin, and 7% (27) to penicillin.

A total of 3617 unique individuals had culture-positive *N. gonorrhoeae* isolates during the study period. Significantly higher frequencies of non-susceptible strains were observed in males for all antimicrobials, except tetracycline, where there was no significant difference noted (Table 1). Penicillin, ciprofloxacin, and azithromycin susceptibility were significantly associated with provincial health zone. The highest frequencies of non-susceptible strains for penicillin and ciprofloxacin were observed in the city of Calgary (92.8% and 56.6%, respectively), and the south zone (90.5% and 52.4%, respectively), while the city of Edmonton and the central zone had the highest proportions of non-susceptibility for azithromycin (6.2% and 8.5%). Individuals from higher income quintiles (Q3, Q4, and Q5) had higher frequencies of non-susceptibility to ciprofloxacin than those in the bottom two quintiles (Q1 and Q2; Table 1), however, the same trend was not seen for other antimicrobials. 

The single isolate resistant to both cefixime and ceftriaxone was from a male 21–30 years old residing in an urban region, with travel history to a country with higher documented rates of cefixime and ceftriaxone AMR [19]. This isolate was identified as the ceftriaxone-resistant multi-locus sequence type 1903/NG-MAST, 3435/NG-STAR 233, which matched the FC428 isolate originally identified in Japan [24,25]. Phenotypic resistance testing was confirmed by sequencing the *penA* gene, which had the same mutations (A311V and T482S) as FC428 and additionally a *penA* mosaic 60.001 marker [19]. Further whole genome sequencing of the Alberta isolate showed clustering with other resistant strains reported from Australia (closest match), Quebec (Canada), and Japan [19]. In a subsequent environmental scan of the concurrent infections in Alberta (within 1.5 months), none of the 232 positive gonococcal cases screened were positive for *penA* mutations, and none matched the NG-MAST-ST-3435 [19].

The case rates for gonorrhea have dramatically increased in Canada over the last decade, with a rate of infection 124% higher in 2021 (32,192 cases) compared to 2012 (13,027 cases) [26]. Interestingly, while in 2012, national case numbers were more equally split between the sexes (7327 males vs. 5682 females), in 2021, a more marked difference was noted (20,258 males vs. 11,821 females) [26]. Similarly, we observed that over 80% of culture-positive *N. gonorrhoeae* cases in Alberta during this period were collected from males, however, our study did not look at total case numbers, but rather the number of samples submitted for culture. Therefore, the higher number of cultures collected from males in our study is likely due to the special populations indicated in local guidelines for gonococcal culture, which include testing for those with treatment failure, suspected AMR in a sexual contact, or for symptomatic individuals who identify as gay, bisexual, or men who have sex with men (gbMSM) [14,27].

In Alberta, the preferred treatment for *N. gonorrhoeae* infection is combination therapy with cefixime and oral azithromycin as a single dose or, alternatively, ceftriaxone (intramuscular) with oral azithromycin [14]. While azithromycin susceptibility remained high throughout the study period, 100% susceptibility was not reached across all years (Figure 1). These findings align with recent national and provincial reports, which highlight rising AMR trends including decreased susceptibility to current treatment options [28,29]. Notably, males exhibited higher frequencies of azithromycin non-susceptibility compared to females (91.77% vs. 8.23%), and gbMSM are recognized as a higher-risk population overall for *N. gonorrhoeae* infection [6].

The AMR trends observed in our study (lowest susceptibility seen in penicillin, tetracycline, and ciprofloxacin) reflect the history of antibiotic use against *N. gonorrhoeae*. With penicillin and tetracycline being longest in use [8], it is unsurprising that these antimicrobials showed the lowest susceptibility year after year. Ciprofloxacin, introduced shortly after tetracycline’s removal from first-line treatment, followed closely in resistance trends [8]. Although emerging resistance to ceftriaxone and cefixime has been reported in other countries [30,31], in Alberta, *N. gonorrhoeae* strains remained 100% susceptible in 2022.

Surveillance programs around the world have identified a growing resistance profile in *N. gonorrhea* strains. The China Gonococcal Resistance Surveillance Program (China-GRSP) reported a significant increase in ceftriaxone resistance, rising from 2.9% in 2017 to 8.1% in 2022, with some provinces reporting resistance levels exceeding 10% [30]. Resistance to cefixime and azithromycin was also reported at 16% and 16.9% respectively [30]. Between 2018 and 2022, the United States Gonococcal Isolate Surveillance Project (GISP) reported 4 cefixime (2 in 2018; 1 in 2019; 1 in 2020) and 1 ceftriaxone (2019) resistant isolates, with similar resistance rates of azithromycin and ciprofloxacin across all years (average 4.8% resistance to azithromycin (range 4.07 to 5.82% and average 33.1% resistance to ciprofloxacin (range 31.02 to 35.42)) [32]. In 2023, a novel *N. gonorrhoeae* strain in the United States exhibited reduced susceptibility to five classes of antibiotics, including ceftriaxone [31]. Similarly, the World Health Organization’s Enhanced Gonococcal Antimicrobial Surveillance Programme (EGASP) reported high levels of resistance in Cambodia to ceftriaxone, cefixime, azithromycin, and ciprofloxacin (15.4%, 43.1%, 14.4% and 97.1%, respectively), with 6.2% of all isolates resistant to all four antimicrobials in 2022–2023 [33]. Similarly, in 2023, the EGASP program in Vietnam identified high levels of resistance to ceftriaxone, cefixime, and azithromycin at 26.9%, 30.9%, and 4.8% respectively [34]. To counteract rising resistance, European and UK treatment guidelines now recommend higher doses of ceftriaxone [35,36]. These developments emphasize the urgent need for alternative treatments to combat AMR.

One promising alternative is zoliflodacin, a DNA biosynthesis inhibitor. In a Phase 2 clinical trial, zoliflodacin was found to be as effective as ceftriaxone for treating urogenital infections and rectal infections, though it was not as effective at treating pharyngeal infections [37]. Another potential treatment option, gepotidacin, which inhibits bacterial DNA replication, recently completed Phase 3 trials for uncomplicated urogenital *N. gonorrhoeae* infections [38,39]. Overall, maintaining up-to-date AMR surveillance can aid in the global fight against antimicrobial resistant *N. gonorrhoeae* and lead to the development of effective treatment options.

Due to *N. gonorrhoeae’s* high antigenic variability, an effective vaccine has not yet been developed. Past trials have examined heat-killed partially lysed gonococci, pilin subunits, and outer-membrane antigens, however, with the exception of one pilin subunit vaccine, which showed protection upon homologous *N. gonorrhoeae* challenge, none offered any protection [40]. Some more recent pre-clinical vaccine candidates have shown efficacy against gonococcal infection in mice, including a combination of a cell division protein (FtsN) with another predicted cell division protein (NGO0265) [41], and a transferrin binding protein (TbpB) [42].

*N. gonorrhoea* and *N. meningitidis* are genetically similar (80–90% genetic homology) [43]; therefore, in 2017, a research group from New Zealand assessed the ability of the outer membrane vesicle meningococcal B vaccine (MeNZB) to provide cross-protection for gonococcal infection [44]. An analysis of nearly 15,000 cases and controls showed that the vaccine was 31% effective at preventing gonococcal disease [44]. This finding was confirmed by others using another outer membrane vesicle vaccine (MenB-4C) with efficacy ranging from 40 to 59% [45,46]. Interestingly, although the numbers are small, a direct comparison of a meningococcal outer membrane vesicle vaccine and a non-OMV vaccine in the same cohort showed the OMV-based vaccine was more effective, with 24 cases of gonococcal disease in the OMV and 44 cases in the non-OMV vaccine [45].

In 2023, a modelling study using the 4CMenB vaccine showed that a national vaccination program against gonorrhea could be highly effective and reduce case number by 50,000 over 10 years. The vaccine, composed of *Neisseria* heparin-binding antigen (NHBA), Neisserial adhesion A (NadA), factor H binding protein (fHbp), and the meningococcal serogroup B outer membrane vesicles (OMVs) [47], has both the OMV and the NHBA, which are displayed on the surface of both *N. meningitidis* and *N. gonorrhoeae*. Based on these data, in June 2025, the United Kingdom announced a new vaccine program to vaccinate high-risk individuals with the meningococcal vaccine (4CMenB, GSK) [48]. This is an exciting step in the reduction of gonococcal disease.

This study was limited by the availability and completeness of the AST data and provincial census data. Individuals not matched back to census data through recorded postal codes were not included in income quintile and geographic region demographic analysis (n = 1956). Additionally, individuals missing data for antimicrobial testing were not included in the denominator for susceptibility results. However, this study represents a comprehensive examination of the AST profiles in Alberta, Canada, in special populations over time.

## 3. Materials and Methods

The government of Alberta (Alberta Health (AH)) recommends gonorrhea testing for symptomatic, high-risk, or pregnant individuals, and retesting following the completion of treatment [27]. Testing every 3 to 6 months is recommended for individuals with a new sexual partner, more than one partner, or anonymous sex partner(s). Through universal health care, testing can be accessed at any STI clinic across the province or through referral by a healthcare provider. Primary diagnostic testing is performed using nucleic acid amplification testing (NAAT) on urine or swab specimens from the vagina, cervix, urethra, rectum, pharynx, or eyes [49]. In cases of treatment failure, suspected AMR in a sexual contact, or for symptomatic individuals who identify as gbMSM, AH recommends additional culture and AST alongside NAATs [27]. Gradient strips (E-test, BioMerieux, Saint-Laurent, QC, USA) are used for susceptibility testing of six antibiotics: azithromycin, cefixime, ceftriaxone, ciprofloxacin, penicillin, and tetracycline per manufacturer’s instructions.

### 3.1. Database Creation and Analysis of AST Results

AST results on culture-positive isolates from Alberta Precision Laboratories—Public Health Laboratory between 2016 and 2022 were extracted from the Millennium and Beaker laboratory information system databases. For AST results, duplicates were removed for individuals with more than one isolate submitted on the same day, under the assumption *N. gonorrhoeae* strains were identical regardless of the sample source. Results were analyzed retrospectively by year based on date of sample collection. Not all isolates underwent AST for each antimicrobial. MICs were defined as susceptible, intermediate, or resistant based on the Clinical and Laboratory Standards Institute (CLSI) M100 guidelines [50].

### 3.2. Database Creation and Analysis of AST Results Stratified by Demographic Variables

Demographic variables were merged into the AST database, and duplicate individuals within the same year were dropped prior to analysis (with the result occurring first in the calendar year kept, and subsequent duplicates from the same individual within that year removed). Age at time of collection was used to group individuals into age categories at 10-year increments as follows: 10–20, 21–30, 31–40, 41–50, 51–60, and >60 years. Provincial health zones were categorized into: Calgary, Central, Edmonton, North, and South zones. Income quintile and geographic region (rural, urban, or metropolitan) were derived from the 2021 Alberta census estimates based on postal codes matched to the AST dataset. Geographic distributions were reported as per Alberta Health’s geography of residence definitions previously reported [51]. Briefly, metro was defined as populations > 500,000, and include the two largest cities in Alberta, Calgary and Edmonton proper; urban areas are populations > 25,000 but less than 500,000 (Grande Prairie, Fort McMurray, Red Deer, Lethbridge, Medicine Hat); rural areas are populations < 25,000 and up to 200 kilometres from a metro or urban centre (these include towns, villages, hamlets, First Nations, Metis Settlements, and agricultural areas); remote areas are considered those areas greater than 200 km from a metro or urban centre. For the purposes of this study, remote and rural were grouped together as ‘rural’ to maintain power for analyses.

Data were compiled and analyzed using STATA v.17 (StataCorp, College Station, TX, USA), and figures were generated using Microsoft Excel (Microsoft Corporation, Redmond, WA, USA). Antimicrobial susceptibility was calculated annually for each antibiotic by dividing the number of susceptible isolates by the total number of isolates tested. Demographic characteristics of patients were compared between those with susceptible and non-susceptible AST results using Chi-squared or Fisher’s Exact tests for small cell counts (<5) with significance set at α ≤ 0.05.

Ethics was approved by the University of Alberta Research Ethics Board Pro00130642, 12 June 2023.

## 4. Conclusions

In conclusion, this study highlights the importance of continuous antimicrobial susceptibility surveillance for *N. gonorrhoeae* in Alberta. Despite the overall high level of susceptibility to ceftriaxone and cefixime, the emergence of non-susceptibility to azithromycin and varying susceptibility patterns across demographic groups underscores the need for ongoing monitoring. The higher rates of non-susceptibility observed for ciprofloxacin and azithromycin in males and for ciprofloxacin for individuals from higher income quintiles suggest potential areas for targeted intervention. These findings emphasize the need for adapting treatment guidelines based on evolving resistance patterns to ensure effective management of gonorrhea and to mitigate the impact of antimicrobial resistance.

## Figures and Tables

**Table 1:** Patient demographics of gonorrhea cases and corresponding AST results for penicillin and tetracycline in Alberta from 2016 to 2022.

**Penicillin (n = 3594)**				**Tetracycline (n = 3592)**				**Ciprofloxacin (n = 3593)**				**Azithromycin (n = 3591)**			
	Susceptible n (%)	Non-Susceptible n (%)	*p*-value *		Susceptible n (%)	Non-Susceptible n (%)	*p*-value *		Susceptible n (%)	Non-Susceptible n (%)	*p*-value *		Susceptible n (%)	Non-Susceptible n (%)	*p*-value *
**Age (years) (n = 3592)**			0.202	**Age (years) (n = 3590)**			0.221	**Age (years) (n = 3591)**			<0.001 *	**Age (years) (n = 3589)**			0.374
10–20 ^a^ (n = 252)	36 (10.68)	216 (6.64)		10–20 ^a^ (n = 251)	76 (7.15)	175 (6.93)		10–20 ^a^ (n = 252)	156 (8.35)	96 (5.57)		10–20 ^a^ (n = 251)	239 (6.97)	12 (7.59)	
21–30 (n = 1520)	139 (41.25)	1381 (42.43)		21–30 (n = 1521)	421 (39.60)	1100 (45.53)		21–30 (n = 1521)	833 (44.59)	688 (39.93)		21–30 (n = 1520)	1463 (42.64)	57 (36.08)	
31–40 (n = 1184)	112 (33.23)	1072 (32.93)		31–40 (n = 1183)	368 (34.62)	815 (32.25)		31–40 (n = 1183)	588 (31.48)	595 (34.53)		31–40 (n = 1183)	1126 (32.82)	57 (36.08)	
41–50 (n = 399)	33 (9.79)	366 (11.24)		41–50 (n = 399)	124 (11.67)	275 (10.88)		41–50 (n = 399)	186 (9.96)	213 (12.36)		41–50 (n = 399)	382 (11.13)	17 (10.76)	
51–60 (n = 191)	15 (4.45)	176 (5.41)		51–60 (n = 191)	65 (6.11)	126 (4.99)		51–60 (n = 191)	91 (4.87)	100 (5.80)		51–60 (n = 191)	177 (5.16)	14 (8.86)	
>60 (n = 46)	2 (0.59)	44 (1.35)		>60 (n = 45)	9 (0.84)	36 (1.43)		>60 (n = 45)	14 (0.75)	31 (1.80)		>60 (n = 45)	44 (1.29)	1 (0.63)	
^‡^ **Sex (n = 3590)**			0.006 *	^‡^ **Sex (n = 3588)**			0.368	^‡^ **Sex (n = 3589)**			<0.001 *	^‡^ **Sex (n = 3587)**			0.001 *
Male (n = 2928)	257 (76.04)	2,671 (82.13)		Male (n = 2928)	877 (82.12)	2051 (81.23)		Male (n = 2928)	1462 (76.38)	1502 (87.22)		Male (n = 2928)	2783 (81.16)	145 (91.77)	
Female (n = 662)	81 (23.96)	581 (17.87)		Female (n = 660)	186 (17.88)	474 (18.77)		Female (n = 612)	441 (23.62)	220 (12.78)		Female (n = 659)	646 (18.84)	13 (8.23)	
**Health Zone (n = 3562)**			<0.001 *	**Health Zone (n = 3561)**			0.092	**Health Zone (n = 3562)**			<0.001 *	**Health Zone (n = 3560)**			0.001 *
Calgary (n = 2039)	146 (43.58)	1893 (58.66)		Calgary (n = 2037)	575 (54.55)	1462 (58.32)		Calgary (n = 2037)	885 (47.73)	1152 (67.45)		Calgary (n = 2037)	1968 (57.85)	69 (43.67)	
Central (n = 59)	8 (2.39)	51 (1.58)		Central (n = 59)	12 (1.14)	47 (1.87)		Central (n = 59)	33 (1.78)	26 (1.52)		Central (n = 59)	54 (1.59)	5 (3.16)	
Edmonton (n = 1330)	159 (47.46)	1,171 (36.29)		Edmonton (n = 1331)	423 (40.13)	908 (36.22)		Edmonton (n = 1332)	845 (45.58)	487 (28.51)		Edmonton (n = 1331)	1249 (36.71)	82 (51.90)	
North (n = 113)	20 (5.97)	93 (2.88)		North (n = 113)	37 (3.51)	76 (3.03)		North (n = 113)	81 (4.37)	32 (1.87)		North (n = 112)	110 (3.23)	2 (1.27)	
South (n = 21)	2 (0.60)	19 (0.59)		South (n = 21)	7 (0.66)	14 (0.56)		South (n = 21)	10 (0.54)	11 (0.64)		South (n = 21)	21 (0.62)	0 (0)	
**Geographic Region (n = 1653)**			0.085	**Geographic Region (n = 1653)**			0.796	**Geographic Region (n = 1654)**			<0.001 *	**Geographic Region (n = 1652)**			0.652
Metro (n = 1478)	144 (85.21)	1334 (89.89)		Metro (n = 1478)	466 (90.14)	1012 (89.08)		Metro (n = 1479)	705 (86.50)	774 (92.25)		Metro (n = 1478)	1413 (89.32)	65 (92.86)	
Urban (n = 50)	5 (2.96)	45 (3.03)		Urban (n = 50)	14 (2.71)	36 (3.17)		Urban (n = 50)	36 (4.42)	14 (1.67)		Urban (n = 49)	47 (2.97)	2 (2.86)	
Rural (n = 125)	20 (11.83)	105 (7/08)		Rural (n = 125)	37 (7.16)	88 (7.75)		Rural (n = 125)	74 (9.08)	51 (6.08)		Rural (n = 125)	122 (7.71)	3 (4.29)	
**Income Quintile (n = 1653)**			0.303	**Income Quintile (n = 1653)**			0.953	**Income Quintile (n = 1654)**			0.002 *	**Income Quintile (n = 1652)**			0.133
Q1 (lowest) (n = 438)	48 (28.40)	390 (26.28)		Q1 (lowest) (n = 438)	137 (26.50)	301 (26.50)		Q1 (lowest) (n = 439)	249 (30.55)	190 (22.65)		Q1 (lowest) (n = 438)	421 (26.61)	17 (24.29)	
Q2 (n = 300)	38 (22.49)	262 (17.65)		Q2 (n = 300)	98 (18.96)	202 (17.78)		Q2 (n = 300)	155 (19.02)	145 (17.28)		Q2 (n = 300)	290 (18.33)	10 (14.29)	
Q3 (n = 300)	25 (14.70)	275 (18.53)		Q3 (n = 300)	95 (18.38)	205 (18.05)		Q3 (n = 300)	135 (16.56)	165 (19.67)		Q3 (n = 300)	287 (18.14)	13 (18.57)	
Q4 (n = 288)	31 (18.34)	257 (17.32)		Q4 (n = 288)	90 (17.41)	198 (17.43)		Q4 (n = 288)	127 (15.58)	161 (19.19)		Q4 (n = 288)	268 (16.94)	20 (28.57)	
Q5 (highest) (n = 327)	27 (15.98)	300 (29.22)		Q5 (highest) (n = 327)	97 (18.76)	230 (20.25)		Q5 (highest) (n = 327)	149 (18.28)	178 (21.22)		Q5 (highest) (n = 326)	316 (19.97)	10 (14.29)	

n: sample size, %: column frequencies, *: *p* < 0.05 is significant (significant values are marked in bold). Chi2 or Fisher’s exact tests used on small cell counts, ^‡^: % tabulated as row totals, ^a^: 5 individuals < 15 years old.

## Data Availability

The aggregate data of non-identifiable patient variables presented in this study are available on request from the corresponding author due to restrictions of the institution providing the dataset (Alberta Precision Laboratories) and the University of Alberta Ethics Board, where approval was granted to allow only aggregate data to be shared to prevent patient identification.
